# Supplementary material for: Prostate Cancer-Specific Lysine 53 Acetylation of Cytochrome c Drives Metabolic Reprogramming and Protects from Apoptosis in Intact Cells
Source: Biomolecules. 2024 Jun 14;14(6):695. doi: 10.3390/biom14060695 (PMC11201891; doi:10.3390/biom14060695)
Supplement: Supplementary file 1 [file biomolecules-14-00695-s001.zip › Supplemental Figures.pdf]

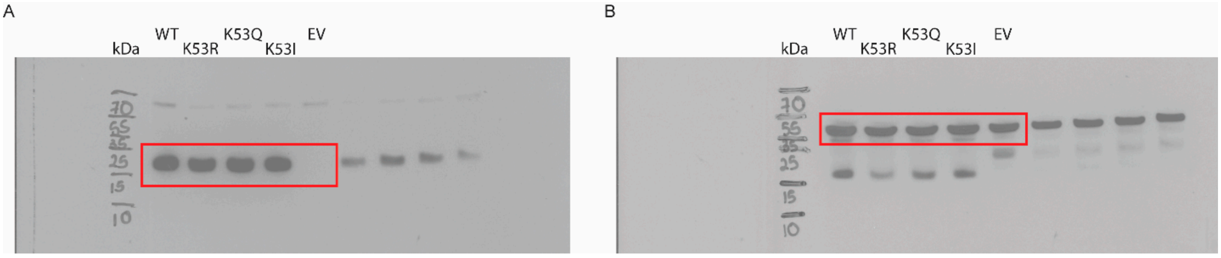

Figure S1. Raw Western Blots for Figure 1A. Raw Western Blot images for **(A)** Cytc and **(B)** GAPDH. The red boxes indicate the areas displayed in Figure 1A.

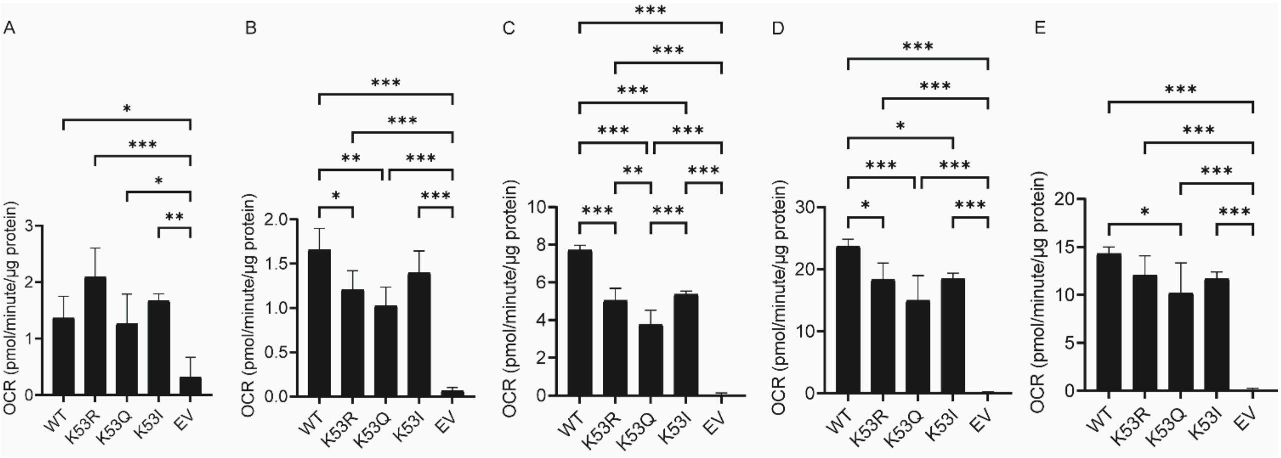

Figure S2. Mitochondrial stress test parameters. **(A)** Non-mitochondrial oxygen consumption, **(B)** proton leak, **(C)** ATP-coupled respiration, **(D)** maximal respiration, and **(E)** spare respiratory capacity of transfected cells (n = 4-5). Data are represented as means ± standard deviation, * $p < 0.05$, ** $p < 0.01$, *** $p < 0.001$.
